# Whole transcriptome analysis and construction of a ceRNA regulatory network related to leaf and petiole development in Chinese cabbage (*Brassica campestris* L. ssp. *pekinensis*)

**DOI:** 10.1186/s12864-023-09239-y

**Published:** 2023-03-24

**Authors:** Fengyan Shi, Zifan Zhao, Yang Jiang, Song Liu, Chong Tan, Chuanhong Liu, Xueling Ye, Zhiyong Liu

**Affiliations:** 1 Department of Horticulture, Shenyang Agricultural University, 120 Dongling Road, Shenhe District, Shenyang, 110866 China; 2 Vegetable Research Institute of Liaoning Academy of Agricultural Sciences, Shenyang, 110161 China

**Keywords:** Chinese cabbage, Leaf, Petiole, Whole transcriptome, ceRNA

## Abstract

**Background:**

The growth and development of leaves and petioles have a significant effect on photosynthesis. Understanding the molecular mechanisms underlying leaf and petiole development is necessary for improving photosynthetic efficiency, cultivating varieties with high photosynthetic efficiency, and improving the yield of crops of which the leaves are foodstuffs. This study aimed to identify the mRNAs, long non-coding RNAs (lncRNAs), microRNAs (miRNAs), and circular RNAs (circRNAs) related to leaf and petiole development in Chinese cabbage (*Brassica campestris* L. ssp. *pekinensis*). The data were used to construct a competitive endogenous RNA (ceRNA) network to obtain insights into the mechanisms underlying leaf and petiole development.

**Results:**

The leaves and petioles of the ‘PHL’ inbred line of Chinese cabbage were used as research materials for whole transcriptome sequencing. A total of 10,646 differentially expressed (DE) mRNAs, 303 DElncRNAs, 7 DEcircRNAs, and 195 DEmiRNAs were identified between leaves and petioles. Transcription factors and proteins that play important roles in leaf and petiole development were identified, including xyloglucan endotransglucosylase/hydrolase, expansion proteins and their precursors, transcription factors TCP15 and bHLH, lateral organ boundary domain protein, cellulose synthase, MOR1-like protein, and proteins related to plant hormone biosynthesis. A ceRNA regulatory network related to leaf and petiole development was constructed, and 85 pairs of ceRNA relationships were identified, including 71 DEmiRNA–DEmRNA, 12 DEmiRNA–DElncRNA, and 2 DEmiRNA–DEcircRNA pairs. Three *LSH* genes (*BrLSH1*, *BrLSH2* and *BrLSH3*) with significant differential expression between leaves and petioles were screened from transcriptome data, and their functions were explored through subcellular localization analysis and transgenic overexpression verification. *BrLSH1*, *BrLSH2* and *BrLSH3* were nuclear proteins, and *BrLSH2* inhibited the growth and development of *Arabidopsis thaliana*.

**Conclusions:**

This study identifies mRNAs and non-coding RNAs that may be involved in the development of leaves and petioles in Chinese cabbage, and establishes a ceRNA regulatory network related to development of the leaves and petioles, providing valuable genomic resources for further research on the molecular mechanisms underlying leaf and petiole development in this crop species.

**Supplementary Information:**

The online version contains supplementary material available at 10.1186/s12864-023-09239-y.

## Background

Leaves are the main photosynthetic organs of plants and they play an important role in plant growth and biomass accumulation. Leaves develop from the shoot apical meristem and the growth and development of leaves is a dynamic process. The transition from small primordia to mature leaves is regulated by two key processes, namely, cell division and expansion [1, 2].

Leaves are divided into two distinct components, the leaf blade and the petiole, which are structurally and physiologically distinct entities [1]. The leaf blade has a wide and flat layer-like structure, in which the palisade and sponge tissue layers absorb incident light. The petiole functions as a stem that physically supports the leaf [3]. To date, few studies have investigated the differences between the two components of leaves (i.e., leaf blades and petioles) and leaf primordia, and the mechanisms underlying the differentiation of leaf blades and petioles have scarcely been explored. Some studies have demonstrated that the cells of leaf blades and petioles originate from a region of proliferation located between these cells. The *AN3* gene plays a key role in maintaining the proliferation of the primordia, and the *bop* gene plays a role in spatial regulation [4]. The development and characteristics of petioles are affected by several factors, including genetic factors, endogenous hormones, and environmental conditions such as light and temperature [5–8].

Certain mRNAs and non-coding RNAs (ncRNAs) related to the development of leaves and petioles have been identified. After the *let* gene was inserted into wild-type *Arabidopsis thaliana*, the petiole of its mutant was subsequently transformed into a leaf, resulting in a leaf-like petiole phenotype [7]. Ge et al. [9] isolated a *MtPHAN* gene insertion mutant using the *Tnt1* retrotransposon of tobacco by using reverse genetic screening technology. *MtPHAN* primarily regulates the development of petioles by regulating cellular elongation. The *Orange* (*Or*) gene in cauliflower (*Brassica oleracea* var*. botrytis*) controls petiolar elongation by inhibiting the expression of the *eRF1* gene [10]. The short petiole character of the SS98206SP line of soybean is regulated by a single recessive gene, *lps3* [11]. Song et al. [12] observed that petiolar elongation in transgenic *A*. *thaliana* plants results from the overexpression of *miR408*, which induces cellular expansion. The overexpression of *miR408* directly or indirectly regulates cytoplasmic growth and gibberellin (GA) biosynthesis and affects a variety of metabolic processes related to photosynthesis, which subsequently alters petiolar phenotypes [12]. Several microRNAs (miRNAs), including *Agr-miR159*, *Agr-miR164*, *Agr-miR166*, *Agr-miR396*, and *Agr-miR408*, are involved in regulating the petiolar development of celery, and it was speculated that the development of leafy petioles could be regulated by their target genes, including the genes encoding the GRF transcription factor, homeodomain leucine zipper (HD-ZIP) transcription factor, TCP transcription factor, and the CUC gene family [13–15]. In *A. thaliana*, the overexpression of *miR396* shortens the period of cell proliferation and reduces the number of cells, which ultimately leads to the formation of small and narrow leaves [16].

Recent studies have demonstrated that the interactions among mRNAs, long non-coding RNAs (lncRNAs), circular RNAs (circRNAs), and miRNAs can affect gene expression, and the competitive endogenous RNA (ceRNA) hypothesis was therefore proposed to explain the mechanisms underlying the regulation of gene expression [17]. Using high-throughput sequencing data obtained from the leaves of *A*. *thaliana*, a previous study identified the circRNAs and lncRNAs associated with the development of leaves, and constructed a ceRNA regulatory network related to leaf development [18]. Huang et al. [19] systematically identified the lncRNAs that are involved in flag leaf senescence of rice, and explored the lncRNA–mRNA regulatory relationships and ceRNA networks during leaf senescence. The circRNAs related to senescence in rice may participate in flag leaf senescence by mediating the expression of parental genes and ceRNA networks [20]. It has been demonstrated that the expression of lncRNAs is tissue-specific in the leaves of pineapple. Co-expression analysis of the lncRNAs and mRNAs in pineapple leaves revealed that the lncRNAs are closely related to photosynthesis genes [21]. Tong et al. [22] systematically identified the circRNAs in the tissues of *Camellia sinensis* at different stages of leaf development by rRNA-depleted circular RNA-seq analysis. The study also determined the structures of circRNAs and obtained novel data pertaining to leaf differentiation during the development of leaf buds into young leaves [22]. However, the ceRNA network related to leaf and petiole development in Chinese cabbage has not been investigated to date.

Chinese cabbage (*B*. *campestris* L. ssp. *pekinensis*) is one of the most important vegetable crops in Asia. The leaves are the main edible parts of Chinese cabbage, and the petioles account for a large proportion of the leaves. The yield of Chinese cabbage is mostly affected by the size of the petioles, and Chinese cabbage is one of the few vegetable crops with expanded petioles that is consumed widely in China. It is therefore necessary to study the molecular mechanisms underlying the development of leaves and petioles in Chinese cabbage. It is noteworthy that genes that promote petiole development may inhibit leaf development to a certain extent, and genes that inhibit petiole development may promote leaf development. Investigating the molecular regulatory mechanisms of petiole development can aid in regulating photosynthetic efficiency, improve the yield of crops with edible leaves, and provide novel strategies for breeding new varieties. In this study, the whole transcriptome of the leaves and petioles of Chinese cabbage was sequenced, and some mRNAs and ncRNAs, including miRNAs, lncRNAs, and circRNAs, that affect the development of leaves and petioles were identified. A ceRNA regulatory network related to leaf and petiole development in Chinese cabbage was constructed for the first time. Additionally, three genes that were significantly differentially expressed between the leaves and petioles were screened by subcellular localization analysis and transgenic overexpression. The findings obtained herein provide a basis for further studies on the regulatory mechanisms underlying leaf and petiole development in Chinese cabbage.

## Results

### Identification and functional enrichment analysis of differentially expressed (DE) mRNAs between leaves and petioles

**Fig. 1:**
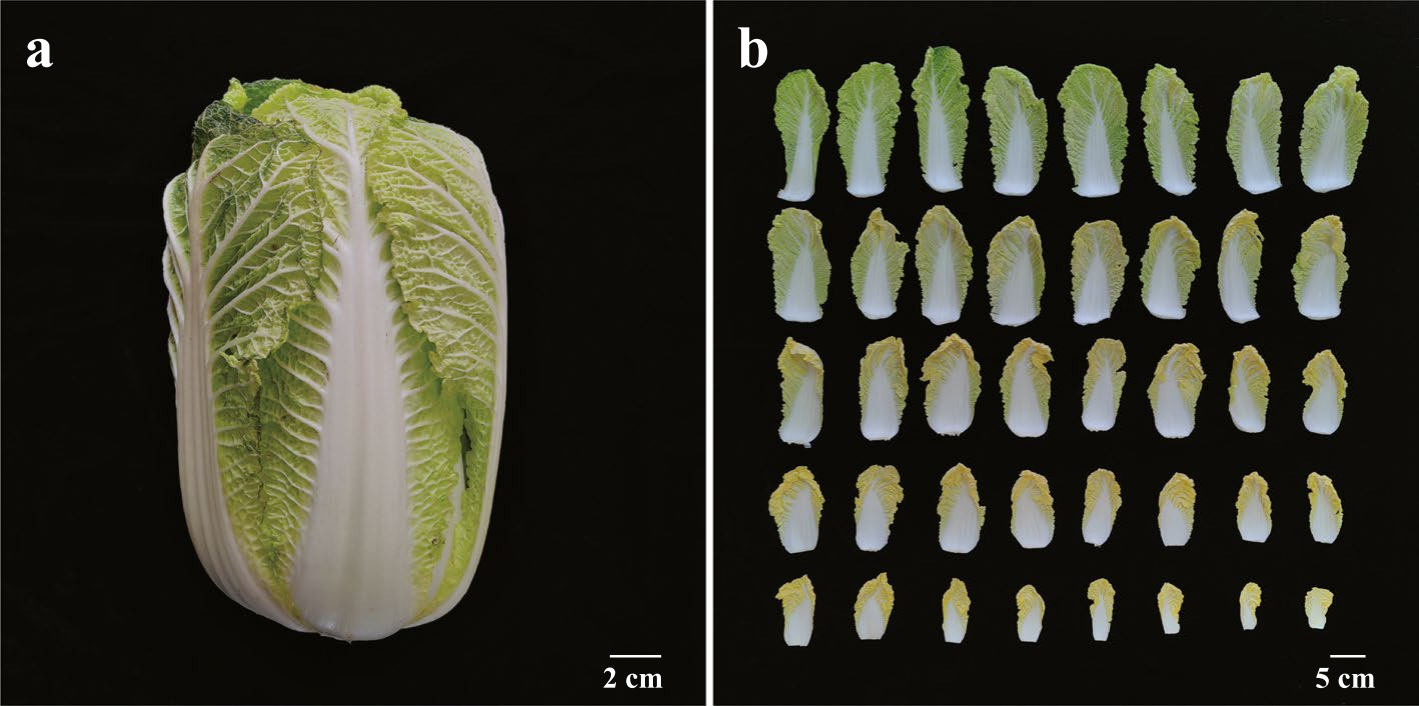
Morphological characteristics of Chinese cabbage. **a** Leafy head; **b** leaf blades and petioles at different developmental stages

**Fig. 2:**
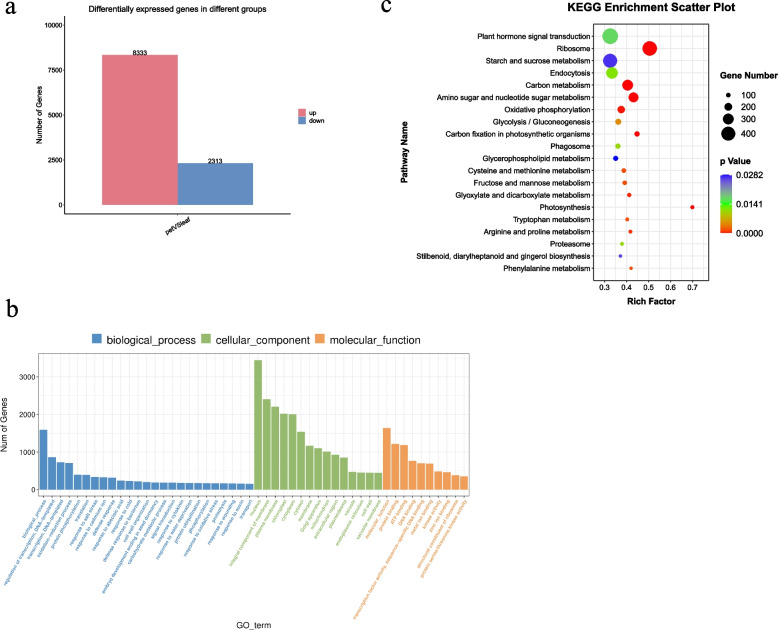
Identification of mRNAs that were differentially expressed between the ‘leaf’ and ‘pet’ samples. **a** Statistical analysis of the DEmRNAs that were upregulated and downregulated between the ‘leaf’ and ‘pet’ samples; **b** GO annotations of the DEmRNAs; **c** KEGG pathway assignments of the DEmRNAs

In this study, two parts of the leaves of Chinese cabbage, namely leaf blades (denoted as ‘leaf’) and petioles (denoted as ‘pet’), were used as the materials for whole transcriptome sequencing analysis on the Illumina NovaSeq 6000 platform (Fig. 1). A total of 370,087,348 and 305,163,478 valid reads were obtained from the ‘leaf’ and ‘pet’ libraries, respectively. The validated ‘leaf’ and ‘pet’ data were mapped to the *B. rapa* v3.0 reference genome, and the mapped rates exceeded 87.31% and 82.53% of the genome, respectively (Table S1). A total of 44,796 mRNAs were identified, and the DEmRNAs were screened using |log_2_ (fold-change)|> 1 and *p* < 0.05 as the standard threshold criteria. A total of 10,646 mRNAs were differentially expressed between the ‘leaf’ and ‘pet’ samples, of which 8333 were upregulated (78.27%) and 2313 were downregulated (21.73%) in ‘pet’ relative to ‘leaf’ (Table S2, Fig. 2a).

**Fig. 3:**
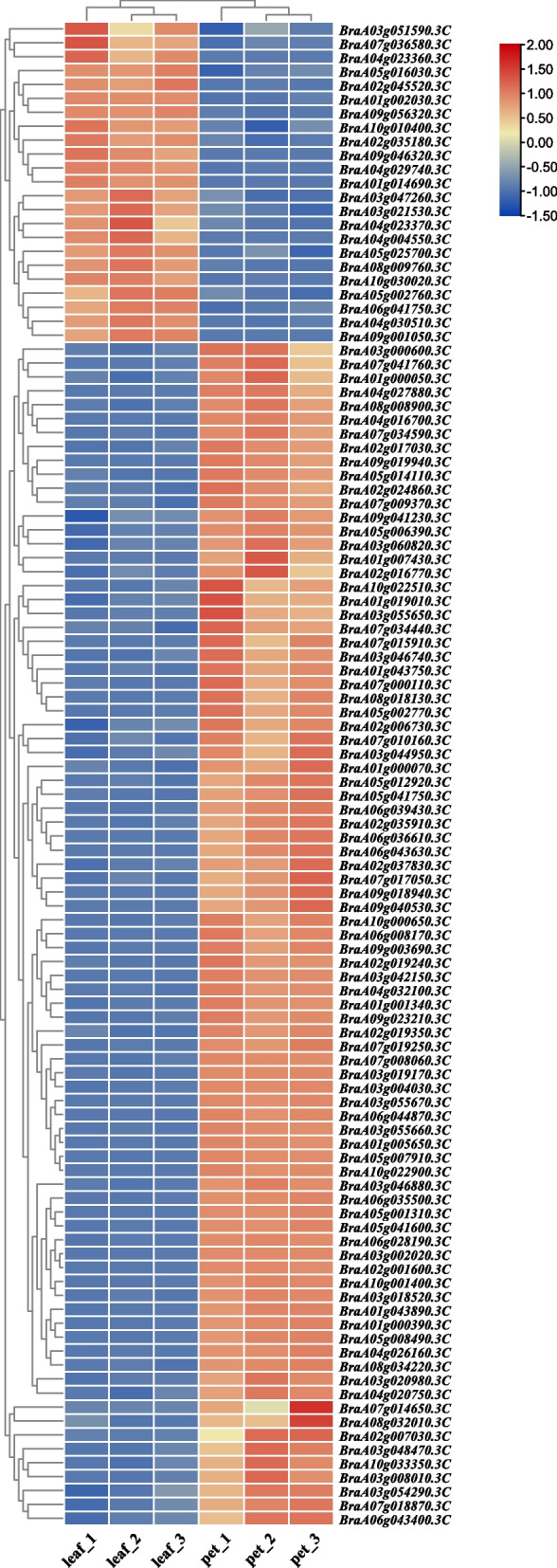
Heatmap of DEmRNAs involved in leaf and petiole development in Chinese cabbage

Some of the DEmRNAs identified herein encoded transcription factors or proteins that may play important roles in the development of leaves and petioles, including xyloglucan endotransglucosylase/hydrolase (XTH), expansion proteins and their precursors, the TCP15 transcription factor, the bHLH transcription factor, lateral organ boundary (LOB) domain protein, cellulose synthase (CESA), MOR1-like protein, and proteins related to plant hormone biosynthesis, including BRASSINAZOLE-RESISTANT1 (BZR1)/BRI1-EMS-suppressor1(BES1) homolog, GA20 oxidase (GA20OX), and DELLA (Fig. 3, Table S3).

The functions of the identified DEmRNAs were determined by Gene Ontology (GO) and Kyoto Encyclopedia of Genes and Genomes (KEGG) enrichment analyses (Tables S4 and S5, Fig. 2b, c). A total of 10,084 DEmRNAs were annotated with 4615 GO terms, and the “nucleus” term was the most enriched. A total of 719 GO terms were significantly enriched at *p* < 0.05, of which 380 (52.9%), 121 (16.8%), and 218 (30.3%) belonged to the biological process, cellular component, and molecular function categories, respectively. The DEmRNAs were mostly enriched in the “biological process,” “oxidation–reduction process,” and “regulation of transcription, DNA-templated” terms under the biological process category; the “nucleus,” “plasma membrane,” and “integral component of membrane” terms under the cellular component category; and the “nucleus function,” “protein binding,” and “ATP binding” terms under the molecular function category. The DEmRNAs were also enriched in several GO terms related to hormone regulation and cell division, including the “response to auxin” and “cell wall” terms, which implied DEmRNAs possibly affect the growth of leaves and petioles. KEGG enrichment analysis revealed that 5940 DEmRNAs were enriched in 138 KEGG pathways, of which 31 were significantly enriched (*p* < 0.05). These significantly enriched pathways were related to leaf development, including “plant hormone signal transduction,” “starch and sucrose metabolism,” and “circadian rhythm.”

### Identification and functional enrichment analysis of DElncRNAs between ‘leaf’ and ‘pet’

**Fig. 4:**
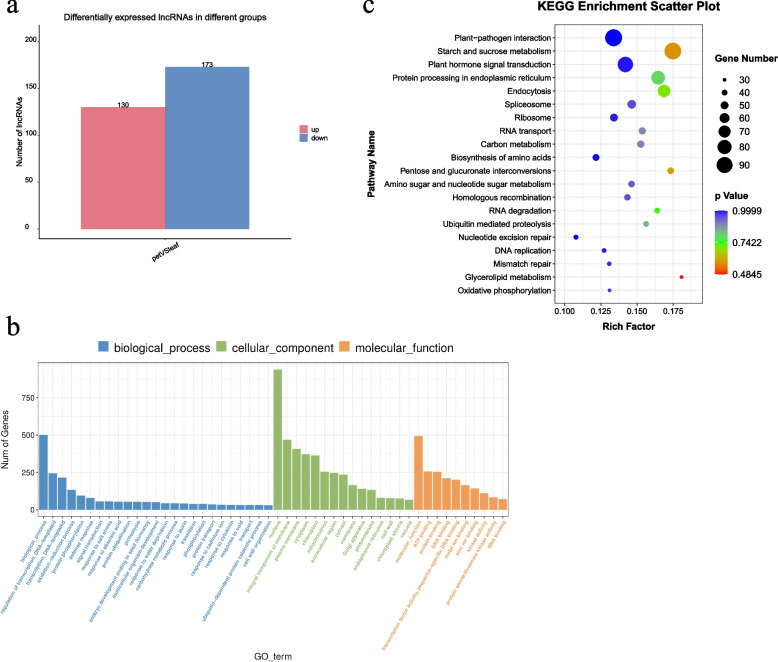
Identification of lncRNAs that were differentially expressed between the ‘leaf’ and ‘pet’ samples. **a** Statistical analysis of the DElncRNAs that were upregulated and downregulated between the ‘leaf’ and ‘pet’ samples; **b** GO annotations of the DElncRNAs; **c** KEGG pathway assignments of the DElncRNAs

A total of 2553 lncRNAs were identified and the DElncRNAs were screened using |log_2_ (fold-change)|> 1 and *p* < 0.05 as the standard threshold criteria. A total of 303 lncRNAs were differentially expressed between the ‘leaf’ and ‘pet’ samples. Compared with the ‘leaf’ samples, a total of 130 (42.9%) and 173 (57.1%) DElncRNAs were upregulated and downregulated, respectively, in the ‘pet’ samples (Table S2, Fig. 4a).

The data revealed that there were significant differences in the structural characteristics and expression levels of lncRNAs and mRNAs (Fig. S1). The majority of mRNA transcripts were > 1000 bp long, while the majority of lncRNA transcripts were < 400 bp (Fig. S1a). The majority of proteins encoded by the open reading frames (ORFs) of the lncRNAs and mRNAs were 0–50 and 100–400 amino acids long, respectively (Fig. S1c, d). The majority of lncRNAs had 1–2 exons, and the exon number of most lncRNAs was lower than that of the mRNAs (Fig. S1b). Additionally, the mRNAs expression levels were higher than the expression levels of lncRNAs (Fig. S1e).

LncRNAs can act as *cis*-regulators of certain adjacent genes and further regulate gene transcription or post-transcriptional gene expression [23]. To explore the *cis*-regulatory functions of the DElncRNAs, a co-expression network comprising 93 DEmRNAs and 63 DElncRNAs was constructed in this study. The results demonstrated that with the exception of eight DElncRNA–DEmRNA pairs which exhibited one-to-one interactions, all the other DElncRNA–DEmRNA pairs exhibited one-to-many interactions (Fig. S2).

The target genes of the DElncRNAs were subjected to GO analysis (Table S4, Fig. 4b). The findings revealed that 2298 target genes were enriched in 2394 GO terms, of which “nucleus,” “biological process,” “molecular function,” and “integral component of membrane” were the most enriched terms. The target genes were significantly enriched in 35 GO terms (*p* < 0.05), consisting of 16 (45.7%), 4 (11.4%), and 15 (42.9%) terms from the biological process, cellular component, and molecular function categories, respectively. The target genes of the DElncRNAs were enriched in “biological process” and “regulation of transcription, DNA-templated” terms under the biological process category; “nucleus,” “integral component of membrane,” and “plasma membrane” terms under the cell component category; and “nucleus function,” “protein binding,” and “ATP binding” terms under the molecular function category.

The results of KEGG analysis demonstrated that 1229 target genes of DElncRNAs were enriched in 127 metabolic pathways (Table S5, Fig. 4c), of which three pathways, namely, “plant–pathogen interaction,” “starch and sucrose metabolism,” and “plant hormone signal transduction,” were the most enriched. These pathways could be involved in hormone and energy regulation during leaf development.

### Identification and functional enrichment analysis of DEcircRNAs between ‘leaf’ and ‘pet’

A total of 886 circRNAs were differentially expressed between the ‘leaf’ and ‘pet’ samples, and a total of 7 DEcircRNAs were identified using the screening criteria |log_2_ (fold-change)|> 1 and *p* < 0.05 (Table S2). Of these, 3 (42.86%) and 4 (57.14%) DEcircRNAs were upregulated and downregulated, respectively, in the ‘pet’ samples compared with the ‘leaf’ samples. The DEcircRNAs identified in this study were unknown and their host genes were not predicted.

### Identification and functional enrichment analysis of DEmiRNAs between ‘leaf’ and ‘pet’

**Fig. 5:**
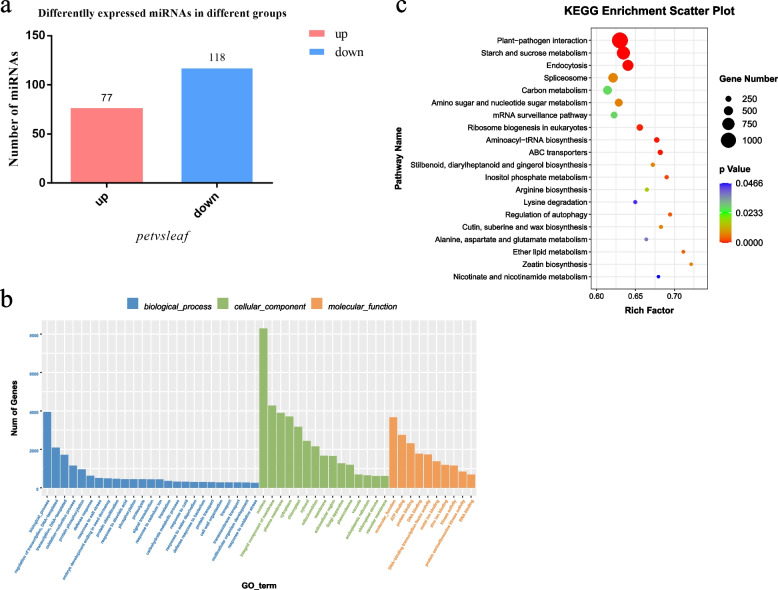
Identification of miRNAs that were differentially expressed between ‘leaf’ and ‘pet’ samples. **a** Statistical analysis of the DEmiRNAs that were upregulated and downregulated between the ‘leaf’ and ‘pet’ samples. **b** GO annotations of the DEmiRNAs; **c** KEGG pathway assignments of the DEmiRNAs

In this study, sRNA libraries of the ‘leaf’ and ‘pet’ samples were constructed and sequenced, aiming to obtain comprehensive insights into the miRNAs related to leaf and petiole development in Chinese cabbage. A total of 8,228,461–9,645,577 valid reads were obtained after screening the sequencing data obtained with an Illumina HiSeq2000/2500 platform. More than 69% of the valid reads could be mapped to pre-miRNAs or reference genomes (Table S6). A total of 1070 miRNAs were identified, of which 195 were differentially expressed between the ‘leaf’ and ‘pet’ samples. A total of 77 (39.49%) and 118 (60.51%) DEmiRNAs were upregulated and downregulated, respectively in the ‘pet’ samples compared with the ‘leaf’ samples (Table S2, Fig. 5a).

The functions of the target genes of the DEmiRNAs were investigated by GO and KEGG enrichment analyses (Tables S4 and S5, Fig. 5b, c). The findings revealed that 20,977 target genes were enriched in 5878 GO terms. The majority of the genes were enriched in the “nucleus,” “plasma membrane,” “cytoplasm,” “ATP binding,” and “cytosol” terms; 544 GO terms were significantly enriched at *p* < 0.05. A total of 277 (50.9%) GO terms in the biological process category were enriched, primarily in the “biological process,” “regulation of transcription, DNA-templated,” and “transcription, DNA-templated” terms. The 67 enriched GO terms of the cellular component category mainly included the terms “nucleus,” “integral component of membrane,” and “plasma membrane”. The 200 enriched GO terms of the biological function category primarily included the “molecular function,” “ATP binding,” and “protein binding” terms. KEGG analysis demonstrated that 12,130 DE target genes of the miRNAs were enriched in 137 KEGG metabolic pathways, of which 24 were significantly enriched (*p* < 0.05). The DE genes were most enriched in the “plant–pathogen interaction” pathway, followed by the “starch and sucrose metabolism” pathway.

### Construction of ceRNA network

**Fig. 6:**
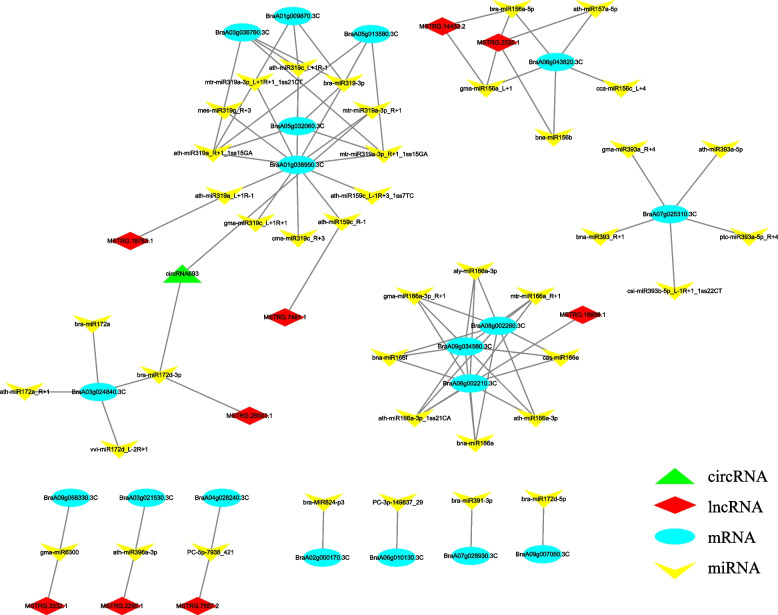
Construction of the ceRNA network related to leaf and petiole development, consisting of DEmRNAs, DElncRNAs, DEcircRNAs, and DEmiRNAs

To explore the regulatory network of ncRNAs and mRNAs related to leaf development, DEmRNAs that may play a key role in leaf development were selected based on the results of gene annotation to establish a ceRNA–miRNA–target gene regulatory network. The ceRNA network comprised 41 DEmiRNAs, 18 DEmRNAs, 9 DElncRNAs, and 1 DEcircRNA. A total of 85 pairs of ceRNA relationships were determined, including 71 DEmiRNA–DEmRNA, 12 DEmiRNA–DElncRNA, and 2 DEmiRNA–DEcircRNA pairs (Table S7, Fig. 6). Analysis of the ceRNA network revealed that most of the miRNAs interacted with circRNAs or lncRNAs, while few miRNAs simultaneously interacted with circRNAs and lncRNAs. It is known that multiple miRNAs can act on the same mRNA and consequently affect gene expression. Functional enrichment analysis of the genes in the ceRNA network revealed that the genes were highly correlated with leaf development. The DE genes were significantly enriched in various GO terms related to leaf development, including “leaf development,” “leaf morphogenesis,” and “cell differentiation” [18]. The majority of DE genes were also significantly enriched in the “plant hormone signal transduction” pathway. The mRNAs and ncRNAs in the ceRNA network can provide insights into the potential molecular mechanisms underlying leaf development or aid in the discovery of novel functional genes.

The ceRNA network comprised several transcription factors and proteins that play an important role in leaf development, including the TCP transcription factors, ATHB protein, and expansin precursor. TCP transcription factors play an important role in cell division and the shape of leaves [24]. The findings revealed that *BraA01g036950.3C* was regulated by *ath-miR159c_L-1R* + *3_1ss7TC*, *ath-miR159c_R-1*, *ath-miR319a_L* + *1R-1*, *ath-miR319a_R* + *1_1ss15GA*, *ath-miR319c_L* + *1R-1*, *bra-miR319-3p*, *cme-miR319c_R* + *3*, *gma-miR319c_L* + *1R* + *1*, *mes-miR319g_R* + *3*, *mtr-miR319a-3p_L* + *1R* + *1_1ss21CT*, *mtr-miR319a-3p_R* + *1*, *mtr-miR319a-3p_R* + *1_1ss15GA*, *circRNA693*, *MSTRG.7481.1*, and *MSTRG.18783.1*; *BraA05g013580.3C* was regulated by *ath-miR319a_R* + *1_1ss15GA*, *bra-miR319-3p*, and *mtr-miR319a-3p_R* + *1_1ss15GA*; *BraA05g032060.3C* was regulated by *ath-miR319a_R* + *1_1ss15GA*, *ath-miR319c_L* + *1R-1*, *bra-miR319-3p*, and *mtr-miR319a-3p_R* + *1_1ss15GA*; *BraA03g036760.3C* was regulated by *ath-miR319a_R* + *1_1ss15GA*, *ath-miR319c_L* + *1R-1*, *bra-miR319-3p*, and *mtr-miR319a-3p_R* + *1_1ss15GA*; and *BraA01g009870.3C* was regulated by *ath-miR319a_R* + *1_1ss15GA*, *ath-miR319c_L* + *1R-1*, and *bra-miR319-3p*.

The ATHB protein belongs to the HD-ZIP transcription factor family and regulates leaf morphology and development of petiolar vascular bundles [25–27]. Our analysis revealed that *BraA06g002210.3C*, *BraA08g002260.3C*, and *BraA09g034560.3C* were regulated by *aly-miR166a-3p*, *ath-miR166a-3p*, *ath-miR166a-3p_1ss21CA*, *bna-miR166a*, *bna-miR166f*, *cas-miR166e*, *gma-miR166a-3p_R* + *1*, *mtr-miR166a_R* + *1*, and *MSTRG.16998.1*.

Expansin proteins and their precursors play an important role in regulating the elasticity of cell walls, which subsequently affects abscission and leaf development [28]. In this study, we observed that *BraA03g021530.3C* was regulated by *ath-miR396a-3p* and *MSTRG.2295.1*.

Network analysis also led to the identification of some mRNAs that were enriched in the “phytohormone signal transduction” KEGG pathway, which could be involved in hormone regulation during leaf development. The results demonstrated that *BraA02g000170.3C* was regulated by *bra-MIR824-p3*; *BraA04g028240.3C* was regulated by *PC-5p-7936_421* and *MSTRG.7557.2*; *BraA06g010130.3C* was regulated by *PC-3p-149837_29*; *BraA06g043820.3C* was regulated by *ath-miR157a-5p*, *bna-miR156b*, *bra-miR156a-5p*, *cca-miR156c_L* + *4*, *gma-miR156a_L* + *1*, *MSTRG.14452.2*, and *MSTRG.2335.1*; *BraA07g025310.3C* was regulated by *ath-miR393a-5p*, *bna-miR393_R* + *1*, *csi-miR393b-5p_L-1R* + *1_1ss22CT*, *gma-miR393a_R* + *4*, and *ptc-miR393a-5p_R* + *4*; *BraA07g028930.3C* was regulated by *bra-miR391-3p*; and *BraA09g058330.3C* was regulated by *gma-miR6300* and *MSTRG.3332.1*.

### Verification by quantitative real-time polymerase chain reaction (qRT-PCR)

The results of RNA-seq analyses were validated by qRT-PCR for 6 mRNAs, 6 lncRNAs, 6 miRNAs, and 6 circRNAs that were differentially expressed between the leaves and petioles of Chinese cabbage (Fig. S3). The mRNA expression data were validated by selecting six genes that could be involved in regulating leaf development. *BraA05g042610.3C* and *BraA02g040480.3C* encode LIGHT-DEPENDENT SHORT HYPOCOTYLS proteins, *BraA07g027780.3C* encodes a bZIP44 transcription factor, *BraA09g034560.3C* encodes the ATHB protein, and *BraA03g042150.3C* and *BraA03g055650.3C* encode XTH proteins. Among the lncRNAs chosen for validation by qRT-PCR, the lncRNAs *MSTRG.16998.1*, *MSTRG.14452.2*, and *MSTRG.11133.1* were present in the ceRNA regulatory network, and *MSTRG.23158.1*, *MSTRG.3458.1*, and *MSTRG.19485.1* were found to be related to hormone regulation. As the functions of the identified circRNAs were unknown, six circRNAs (*circRNA58*, *circRNA601*, *circRNA162*, *circRNA693*, *circRNA612*, and *circRNA136*) were randomly selected from the results of RNA-seq for validation by qRT-PCR. A total of six miRNAs were selected for qRT-PCR validation of RNA-seq data, of which four (*bra-miR319-3p*, *bra-miR156a-5p*, *bra-miR391-3p*, and *vvi-miR172d_L-2R* + *1*) were present in the ceRNA networks. The results of the qRT-PCR analyses were consistent with the results of RNA-seq analyses, which confirmed the reliability of the RNA-seq data.

### Subcellular localization of *BrLSH1*, *BrLSH2*, and *BrLSH3*

Zhao et al. [29] observed that the function of *LSH1* depends on photoregulation during seedling development, which is mediated by phytochromes. Overexpression of *LSH1* results in the production of shorter hypocotyls and larger cotyledons in varying light conditions, and the petioles of *A*. *thaliana* plants overexpressing *LSH1* are smaller than those of the wild-type [29]. Additionally, Lee et al. [30] demonstrated that the overexpression of *AtLSH1* and *AtLSH2* markedly reduces vegetative and reproductive growth in *A*. *thaliana*; however, the phenotypes of *AtLSH1* and *AtLSH2* knockout lines are not significantly different from those of the wild-type. The function of *LSH* genes in Chinese cabbage has not been explored. In this study, three *LSH* genes, namely, *BrLSH1*, *BrLSH2*, and *BrLSH3*, were screened from the transcriptome data, and the expression levels of these genes were found to be significantly higher in the petioles of Chinese cabbage than in the leaves. We speculate that *LSH* genes may affect the development of leaves and petioles in Chinese cabbage. Therefore, in order to further explore their functions, subcellular localization and overexpression transgenic verification were carried out.

**Fig. 7:**
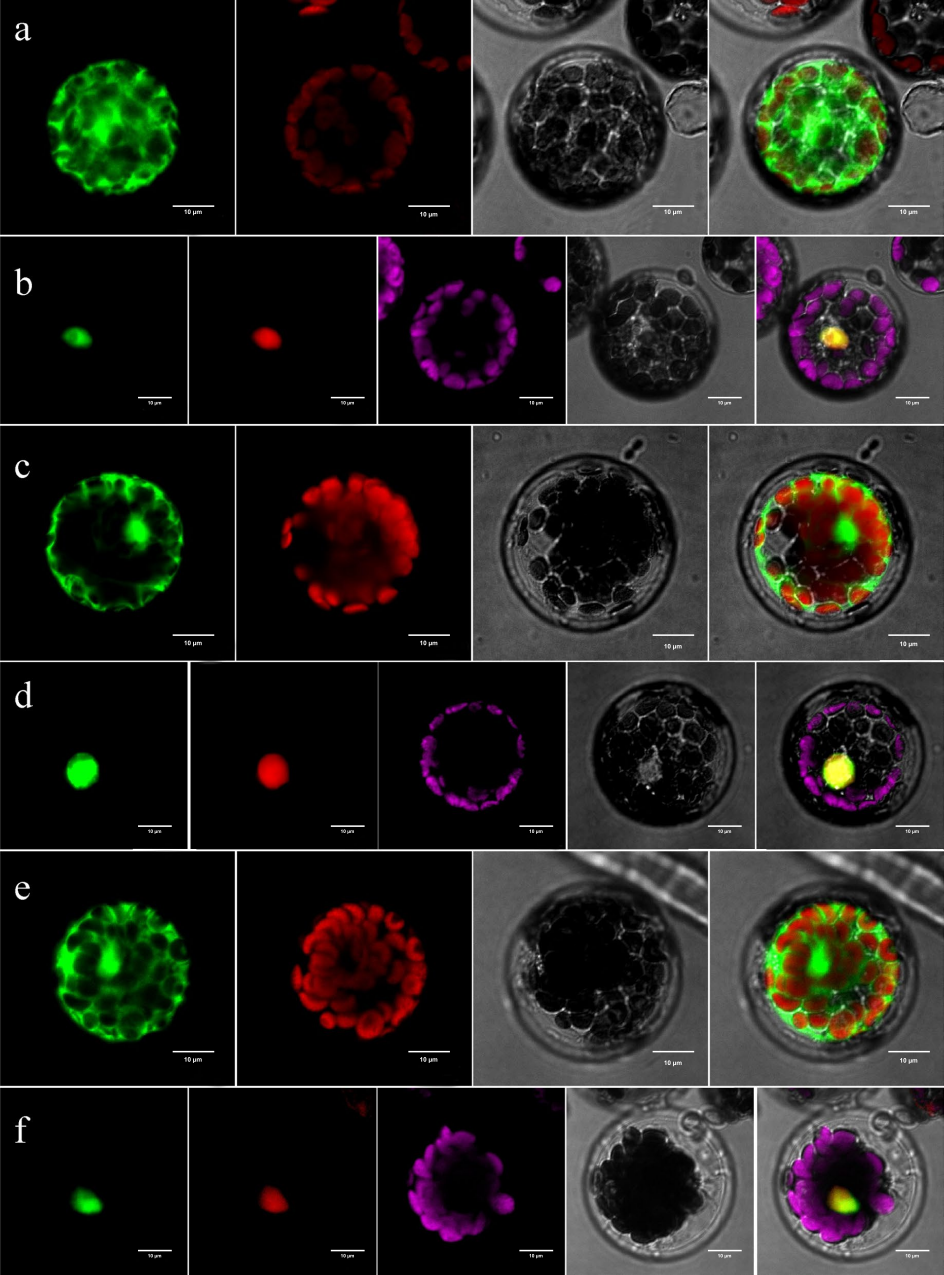
Subcellular localization of *BrLSH1* (**a**, **b**), *BrLSH2* (**c**, **d**), and *BrLSH3* (**e**, **f**). Note: Panels **a**, **c**, and **e** depict the empty carrier fluorescence channel, chloroplast fluorescence channel, bright field, and superposition diagram from left to right; Panels **b**, **d**, and **f** depict the fluorescence channel of the target protein, marker fluorescence channel, chloroplast fluorescence channel, bright field, and superposition diagram from left to right

Here, the subcellular localization of three *LSH* genes, namely, *BrLSH1*, *BrLSH2*, and *BrLSH3*, which were significantly differentially expressed between the ‘leaf’ and ‘pet’ samples of Chinese cabbage, was analyzed. The GFP overexpression vectors PBWA(V)HS1-3-GLosgfp, PBWA(V)HS2-2-GLosgfp, PBWA(V)HS6-3-GLosgfp, and mKATE, were co-transferred into mesophyll cell protoplasts of *A*. *thaliana* by PEG-mediated transformation, and the GFP signals of *BrLSH1*, *BrLSH2*, and *BrLSH3* were observed using a laser scanning microscope. The results demonstrated that *BrLSH1*, *BrLSH2*, and *BrLSH3* were localized in the nucleus, which indicated that *BrLSH1*, *BrLSH2*, and *BrLSH3* encode nucleoproteins (Fig. 7).

### Phenotypic effects of *BrLSH1*, *BrLSH2*, and *BrLSH3*

**Fig. 8:**
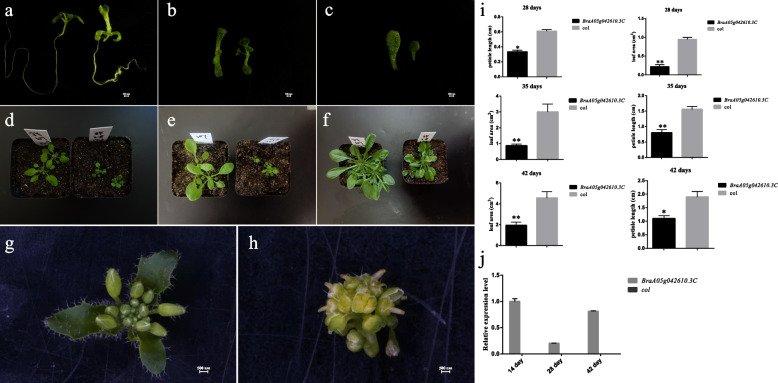
Morphological characteristics of plants overexpressing *BrLSH2*.** a**–**c** Phenotypic differences between wild-type *col* plants (left) and *A. thaliana *plants overexpressing *BrLSH2* (right) 14 days after sowing. **d**–**f** Phenotypic differences between the wild-type *col* plants (left) and *A. thaliana *plants overexpressing *BrLSH2 *(right) 28, 35, and 42 days after sowing. Flower buds of **g** wild-type *A.thaliana* and **h** plants overexpressing *BrLSH2. i* Statistics for the leaf area and petiole length of wild-type plants and *A. thaliana *overexpressing *BrLSH2*.* j* Expression levels of *BrLSH2 *in wild-type (*col*) plants and plants overexpressing *BrLSH2* at different developmental stages

To further explore the functions of *BrLSH1*, *BrLSH2* and *BrLSH3*, the genes were overexpressed in wild-type Col-0 *A*. *thaliana* and the phenotypic effects were observed. The phenotypes of the plants with *BrLSH1* and *BrLSH3* overexpression did not differ significantly from that of wild-type plants. However, the phenotype of plants with *BrLSH2* overexpression altered significantly 14 days after sowing compared with that of the wild-type plants. The plants overexpressing *BrLSH2* had shorter hypocotyls, significantly elongated true leaves, curly cotyledons, shorter taproots, and more lateral roots (Fig. 8a–c). Additionally, the growth rate of the plants overexpressing *BrLSH2* was slightly lower than that of the wild-type plants, especially after 28, 35, and 42 days (Fig. 8d–f). The growth of plants with *BrLSH2* overexpression was significantly inhibited, and the length of the petioles and leaf area were significantly reduced compared with those of wild-type plants (Fig. 8i). The leaf buds underwent division and the petals were degenerated at the bolting stage in the inflorescence of plants overexpressing *BrLSH2* (Fig. 8g, h). These findings indicated that the overexpression of *BrLSH2* had an obvious inhibitory effect on the growth and development of *A*. *thaliana*.

The expression pattern of *BrLSH2* at different stages of development was determined by qRT-PCR analysis of samples of *A*. *thaliana* plants overexpressing *BrLSH2* (Fig. 8j). The results demonstrated that the expression of *BrLSH2* was zero in wild-type plants without exogenous gene transfer, and the expression of *BrLSH2* was evident in plants overexpressing *BrLSH2.* The expression of *BrLSH2* was increased significantly in the seedling stage 14 days after sowing and at the commencement of the bolting stage 42 days after sowing, compared with that 28 days after sowing.

## Discussion

Leaves produce energy and carbohydrates by photosynthesis to maintain plant growth. The development of leaves has been studied in several plants, especially *A*. *thaliana*. The size, shape, and differentiation of the leaves of *A*. *thaliana* are regulated by several genes, including genes encoding mRNAs and miRNAs [16, 31]. Although the regulatory mechanism underlying leaf development is being studied increasingly, the mechanisms of regulation of leaf development in Chinese cabbage remain unclear, and a complete ceRNA regulatory network related to leaf and petiole development has not been constructed to date. In this study, the high generation inbred “PHL” line of Chinese cabbage was selected based on the growth period and genetic background, and leaves and petioles were collected for sequencing and whole transcriptome analyses. A total of 10,646 mRNAs, 303 lncRNAs, 195 miRNAs, and 7 circRNAs of unknown function were identified in this study that were differentially expressed between the leaves and petioles. This study is the first to construct a ceRNA–miRNA–target gene regulatory network related to leaf and petiole development in Chinese cabbage, which can provide a basis for further studies on the molecular mechanisms underlying leaf and petiole development.

GO and KEGG enrichment analyses were performed to elucidate the functions of genes that were differentially expressed between the leaves and petioles of Chinese cabbage. GO enrichment analysis revealed that the majority of DE genes were enriched in the terms “membrane,” “plasma membrane,” and “cell wall.” Additionally, several GO terms related to hormone synthesis, cell proliferation, and expansion were identified, including “brassinosteroid-mediated signaling pathway,” “auxin-activated signaling pathway,” “growth regulation,” “cell proliferation,” and “leaf development”. The results of KEGG enrichment analysis revealed that the majority of DE genes were involved in “plant hormone signal transduction” and “starch and sucrose metabolism” pathways, which are involved in hormone regulation and energy supply during leaf development [32, 33]. These findings may aid in exploring the upstream and downstream genes of functional genes, which can provide further insights into the molecular mechanisms of leaf and petiole development in Chinese cabbage.

The cell wall is a unique structure of plant cells, and the expansion of plant cells depends on the relaxation of cell walls to increase water absorption by the cells. This process requires the involvement of several genes, including those encoding XTHs and expansins [34–37]. Some studies have demonstrated that petiolar elongation is closely related to the modification of the cell wall [38, 39]. In this study, 16 genes encoding XTHs (including 14 upregulated and two downregulated genes in ‘pet’ relative to ‘leaf’), and 27 genes encoding expansion proteins and their precursors (including 23 upregulated genes and four downregulated genes in ‘pet’ relative to ‘leaf’) were identified in the transcriptome data. These genes may participate in cell expansion and affect the development of leaves and petioles. The transcription factors TCP14 and TCP15 participate in the elongation of petioles and hypocotyls. These transcription factors inhibit the transition from mitosis to endoreduplication and promote cellular proliferation, and are inhibited by the negative growth regulator *DA1* [40, 41]. In this study, *BraA07g034590.3C* and *BraA02g019350.3C* were predicted to encode the TCP15-like transcription factor. These genes were upregulated in ‘pet’ samples and may participate in petiolar elongation in Chinese cabbage.

The bHLH transcription factors of plants are involved in a wide range of activities, including stress response, flowering, cell proliferation, and expansion [42]. For instance, *HBI1* encodes a bHLH transcription factor that directly regulates the expression of genes involved in cellular expansion, including the genes encoding the TCP14 and TCP15 transcription factors [43]. In this study, a total of 23 genes encoding bHLH transcription factors were identified, of which 8 and 15 genes were downregulated and upregulated, respectively, in the ‘pet’ samples compared with the ‘leaf’ samples.

A previous study demonstrated that the *MtPHAN* gene of *Medicago truncatula* inhibits the ectopic expression of *ELONGATED PETIOLULE1 (ELP1*), which encodes a LOB domain protein and subsequently promotes the elongation of petiolar cells, which alters the petiolar phenotype [9]. A total of 11 genes encoding LOB domain proteins were identified in our transcriptome data. These genes included *BraA02g045520.3C* which was downregulated in ‘pet’ compared with ‘leaf,’ and *BraA07g017050.3C*, *BraA02g037830.3C*, *BraA03g044950.3C*, *BraA01g001340.3C*, *BraA05g014110.3C*, *BraA02g016770.3C*, *BraA03g060820.3C*, *BraA07g018870.3C*, *BraA07g010160.3C*, and *BraA09g041230.3C*, which were upregulated in the ‘pet’ samples compared with the ‘leaf’ samples. These genes possibly affect the size of the petioles of Chinese cabbage.

Brassinosteroid (BR) is an endogenous plant hormone that is involved in several aspects of plant growth and development, and also regulates cellular expansion. Exogenous BRs promote the elongation of petioles in carrot by affecting cellular elongation, GA content, and cellulose deposition. DELLA proteins act as mediators between BRs and GAs. BES1 and BZR1 are two key transcription factors that are involved in BR biosynthesis and the regulation of plant growth [44]. In this study, three genes encoding BES1/BZR1 homolog proteins were identified, including *BraA01g002030.3C*, which was downregulated in ‘pet’, and *BraA07g041760.3C* and *BraA02g024860.3C*, which were upregulated in ‘pet’ compared with ‘leaf.’ Some studies have demonstrated that GAs play a key role in the proliferation and expansion of petiolar cells [45, 46]. *GA20OX* is a key gene for GA biosynthesis, and DELLA proteins such as GAI, RGA, RGL1, RGL2, and RGL3 inhibit the expression of related genes [47–50]. In this study, *BraA10g010400.3C* and *BraA10g030020.3C*, which encode GA20OX proteins, were downregulated in ‘pet’ samples, while *BraA02g017030.3C*, *BraA09g023210.3C*, and *BraA10g022510.3C*, which encode three DELLA proteins, RGA2, RGL1, and RGL3, were upregulated in the ‘pet’ samples, compared with ‘leaf’ samples.

Cellulose synthesis is regulated by CESA, and the overexpression of *CESA* promotes cellular elongation [44]. In this study, a total of 20 genes related to *CESA* were differentially expressed between the ‘leaf’ and ‘pet’ samples, of which 15 genes were upregulated and 5 were downregulated in the ‘pet’ samples. Cortical microtubules (cMTs) are special cytoskeletal structures that regulate the growth of plant cells. cMTs can regulate the expression of cell wall-modifying proteins by controlling auxin distribution, which mediates the elongation of petioles. MOR1 is a microtubule-associated protein that plays an important role in the organization of cMTs [39]. In this study, we predicted that *BraA03g018520.3C* encodes a MOR1-like protein, and it was significantly upregulated in ‘pet’ compared with ‘leaf.’

MiRNA-based ceRNA network systems are increasingly being established with the rapid development of second-generation sequencing technologies. The ceRNA theory transforms the independent individual relationships of coding and ncRNAs into an interconnected, complex regulatory network. MiRNAs act as core nodes in ceRNA networks, and not only regulate the expression of circRNAs and lncRNAs by competitive combination, but also regulate the expression of mRNAs. Although the three types of ncRNA (miRNAs, circRNAs, and lncRNAs) are not translated into proteins, they affect mRNA translation via competitive binding [51]. Here, to further investigate the functions of ncRNAs related to leaf development, we initially constructed a ceRNA network, which included 42 miRNAs, 19 mRNAs, 9 lncRNAs and 1 circRNA; the RNAs in this ceRNA regulatory network were significantly differentially expressed between the ‘leaf’ and ‘pet’ samples. Previous studies have identified several miRNAs that regulate the development of plant leaves. The *miR396* miRNA of *A*. *thaliana* prevents cell proliferation and inhibits the expression of GRFs, which leads to the formation of small, narrow leaves [52]. Additionally, *miR319* regulates the development of compound leaves in tomato [53]. It has been reported that the transient expression of several genes related to leaf development in rice is affected by *miR156* [54]. The *miR166* miRNA of celery targets the HD-ZIP transcription factor to alter vascular development [13]. In this study, we identified that *BraA03g021530.3C*, which encodes an expansin precursor, was the target gene of *ath-miR396a-3p* and interacted with *MSTRG.2295.1*. Our findings also revealed that five TCP transcription factors, *BraA01g036950.3C*, *BraA05g013580.3C*, *BraA05g032060.3C*, *BraA03g036760.3C*, and *BraA01g009870.3C*, were regulated by *miR319*, and interacted with *MSTRG.18783.1* and *circRNA396*. The *BraA06g043820.3C* gene, which was upregulated in ‘pet’, was the target gene of *bna-miR156b*, *bra-miR156a-5p*, *ca-miR156c_L* + *4*, and *gma-miR156a_L* + *1*, and interacted with *MSTRG.14452.2* and *MSTRG.2335.1*. *BraA06g002210.3C*, *BraA08g002260.3C*, and *BraA09g034560.3C*, which encode HD-ZIP transcription factors, interacted with *MSTRG.16998.1*, and were regulated by the miRNAs *aly-miR166a-3p*, *ath-miR166a-3p*, *ath-miR166a-3p_1ss21CA*, *bna-miR166a*, *bna-miR166f*, *cas-miR166e*, *gma-miR166a-3p_R* + *1*, and *mtr-miR166a_R* + *1.* Further studies on the roles of these mRNAs and ncRNAs in leaf development would provide novel insights into the molecular mechanisms underlying leaf development in Chinese cabbage.

*LSH* genes exist extensively in *Brassica*; the majority of *LSH* genes are highly expressed in petioles, and they may affect leaf morphology [55]. *BrLSH1*, *BrLSH2*, and *BrLSH3* belong to the ALOG family, which exists in most plants, but their functions have not been clearly elucidated. In this study, the results of subcellular localization analyses revealed that *BrLSH1*, *BrLSH2*, and *BrLSH3* were localized to the nucleus. The phenotypes of *A*. *thaliana* plants overexpressing *BrLSH1* and *BrLSH3* did not differ significantly from those of wild-type *A*. *thaliana*. However, the growth of plants overexpressing *BrLSH2* was significantly inhibited, the hypocotyls were shortened, and the length of the petioles and leaf area were significantly reduced, indicating that *BrLSH2* overexpression significantly inhibited plant growth and development. The *BrLSH2* gene is homologous to the *AtLSH2* gene of *A*. *thaliana*. Some studies have demonstrated that the overexpression of *AtLSH2* inhibits the growth and reproductive ability of plants, and that DE *AtLSH2-OX* genes are significantly enriched in stress-related GO terms [30]. These findings indicate that *BrLSH2* may play an important role in biotic and abiotic stresses during the development of leaves and petioles in Chinese cabbage, and further studies are necessary to verify the functions of *BrLSH2*.

## Conclusions

In this study, whole transcriptome sequencing was performed using leaf and petiole samples of Chinese cabbage. A total of 10,646 mRNAs, 303 lncRNAs, 7 circRNAs, and 195 miRNAs were found to be differentially expressed between the ‘leaf’ and ‘pet’ samples. GO and KEGG pathway enrichment analyses of the DE RNAs revealed their important roles in regulating leaf and petiole development. The study also identified genes that encode transcription factors and proteins which play important roles in leaf and petiole development, including genes encoding XTH, expansion proteins and their precursors, TCP15 and bHLH transcription factors, a LOB domain protein, CESA, MOR1-like protein, and proteins related to plant hormone biosynthesis. This study was the first to construct a ceRNA–miRNA–target gene regulatory network related to leaf and petiole development in Chinese cabbage. The network contained 41 DEmRNAs, 18 DEmiRNAs, 9 DElncRNAs, and 1 DEcircRNA. Subcellular localization analysis and transgenic overexpression of *BrLSH1*, *BrLSH2* and *BrLSH3*, which were differentially expressed between the leaves and petioles, were also performed. The results demonstrated that *BrLSH1*, *BrLSH2*, and *BrLSH3* were nucleoproteins, and *BrLSH2* overexpression significantly inhibited the growth and development of *A*. *thaliana*. Our findings provide a systematic recognition of mRNAs and ncRNAs related to leaf and petiole development, and serve as a basis for further exploration of the molecular mechanisms underlying the regulation of leaf and petiole development in Chinese cabbage.

## Methods

### Plant materials

The research material for this study was Chinese cabbage inbred line ‘PHL’ independently created by our laboratory (Liaoning Key Laboratory of Cruciferae Vegetable Crop Genetics and Breeding). The seeds were first germinated in a Petri dish on a wet filter paper at a room temperature (22–25℃), and subsequently sown in the greenhouse of Shenyang Agricultural University. The leaves and petioles were sampled at maturity of Chinese cabbage, and leaves and petioles at different development stages were uniformly mixed together to produce the samples ‘leaf’ and ‘pet’, respectively (Fig. 1). The two groups of samples were frozen in liquid nitrogen and stored at − 80℃. Each group had three biological replicates.

### Library construction and RNA sequencing

The total RNA of the ‘leaf’ and ‘pet’ samples was isolated and purified using Trizol reagent (Invitrogen, CA, USA). The content and quality of the RNA were assessed using a NanoDrop ND-1000 spectrophotometer (Wilmington, DE, USA) and an Agilent 2100 Bioanalyzer (CA, USA).

To analyze the expression of mRNAs, lncRNAs, and circRNAs, a Ribo-Zero™ rRNA Removal Kit (Illumina, San Diego, USA) was used to remove ribosomal RNA for construction of the chain-specific library. Each of the ‘pet’ and ‘leaf’ samples comprised three biological replicates, and hence a total of six libraries (‘leaf 1,’ ‘leaf 2,’ ‘leaf 3,’ ‘pet 1,’ ‘pet 2,’ and ‘pet 3’) were prepared. The qualified libraries were sequenced using the Illumina NovaSeq™ 6000 system (LC-BIO, China). The read length of the double-ended sequence was 2 × 150 bp.

A total of six small RNA libraries was prepared using TruSeq Small RNA Sample Prep Kits (Illumina, San Diego, USA) to analyze miRNA expression. The prepared libraries were sequenced using the Illumina HiSeq 2000/2500 system (LC-BIO, China). The single sequence read length was 1 × 50 bp.

### Identification and analysis of DEmRNAs, DElncRNAs, DEcircRNAs, and DEmiRNAs

The raw reads were processed using the Cutadpt software to remove reads that contained undetermined bases, adapters, or low-quality sequencing data [56]. The quality of the data was verified using the FastQC tool (http://www.bioinformatics.babraham.ac.uk/projects/fastqc/). Valid data was mapped to the reference genome of *B*. *rapa* V3.0 (http://brassicadb.cn/#/SearchJBrowse/?Genome=Brara_Chiifu_V3.0) using Hisat [57].

The StringTie and edgeR programs were used for determining mRNA expression levels to analyze variations in mRNA transcripts [58, 59]. DEmRNAs were screened using |log_2_ (fold-change)|≥ 1 and *p* < 0.05 as the threshold criteria. The GO database and the Blast2GO software were used for GO enrichment analysis [60]. The KEGG pathway database (www.kegg.jp/kegg/kegg1.html) was used for KEGG pathway analysis [61].

The StringTie tool was used to process the transcriptome, and the Coding Potential Calculator and Coding-Non-Coding Index were used for lncRNA prediction [62–64]. The *cis*-target genes of lncRNAs were predicted to help determine their functions. DElncRNAs were selected using |log_2_ (fold-change)|≥ 1 and *p* < 0.05 as the threshold criteria.circRNAs were identified using CIRCExploter2 and CIRI tools [63, 65]. The criteria for identifying circRNAs were based on the splicing sequence and structural characteristics of the circRNAs, as previously described by Shi et al. [66]. DEcircRNAs were selected using |log_2_ (fold-change)|≥ 1 and *p* < 0.05 as the threshold criteria.

The miRNA data were processed using the ACGT101-miR program (LC Sciences, Houston, Texas, USA), and analyzed as follows: (1) acquisition of clean data: reads containing 3′- adaptors and garbage reads were removed; (2) selection of read length: reads of length 18–25 nucleotides were retained; (3) read mapping: the remaining reads were mapped to mRNA, RFam, and Repbase data; and (4) miRNA identification: the valid data were mapped to the pre-miRNAs and reference genome. DEmiRNAs were selected using |log_2_ (fold-change)|≥ 1 and *p* < 0.05 as the threshold filtering criteria. Target genes of miRNAs were predicted using CleaveL and 4:GSTAr [51]. The miRNA targets were subsequently subjected to GO and KEGG enrichment analyses.

### Construction of ceRNA network

ceRNA analysis was divided into two parts, namely, prediction of miRNA–mRNA pairs and prediction of miRNA–lncRNA/circRNA pairs. The Targetfinder software was used for predicting miRNA–mRNA pairs, and the degree of match between the target genes and miRNAs was used as the screening criterion [67]. The Ssearch36 software v36.3.6 and target mimics were used for screening and predicting miRNA–lncRNA/circRNA pairs [68, 69]. Perl scripts were used for integrating the circRNA/lncRNA–miRNA–mRNA networks, and the regulatory relationships were visualized with Cytoscape (https://cytoscape.org) [70]

### qRT-PCR validation

A Trizol kit (Invitrogen, USA) and Evo M-MLV RT Kit II (Accurate Biotechnology, AG11711, China) were used to extract total RNAs of ‘leaf’ and ‘pet’ samples, which were reverse-transcribed into cDNA. Oligo dT and random 6-mer primers were used for reverse transcription of the lncRNAs and mRNAs. Reverse transcription of the circRNAs was performed using downstream primers in qRT-PCR and random 6-mer primers. The reverse transcription of miRNA was performed using primers downstream of U6 and specific stem-loop primers. A SYBR® Green Premix Pro Taq HS qPCR Kit (Accurate Biotechnology) was used to perform qRT-PCR using an ABI 7300 RT-PCR system (Thermo Fisher Scientific, Waltham, MA, USA). The protocols and conditions for qRT-PCR have been described previously, by Shi et al. [66]. The miRNA expression levels were normalized to those of U6 miRNA, which was used as the endogenous control. The expression levels of the mRNAs, lncRNAs, and miRNAs were normalized to those of *actin*. The primer sequences used for RT-PCR are listed in Table S8. qRT-PCRs were performed with three technical replicates and four biological replicates.

### Analysis of subcellular localization

To determine the functions and expression patterns of genes that were possibly involved in leaf development, three ALOG family genes, namely *BraA03g031670.3C*, *BraA05g042610.3C*, and *BraA02g040480.3C* (referred to as *BrLSH1*, *BrLSH2*, and *BrLSH3*, respectively), which were significantly upregulated in the ‘pet’ samples relative to the ‘leaf’ samples were selected for subcellular localization analysis. The full-length coding sequences of *BrLSH1*, *BrLSH2*, and *BrLSH3* were amplified using the genomic cDNA of Chinese cabbage. The primers used for amplification are listed in Table S9, where the uppercase letters indicate *Aar*I restriction sites. The products of PCR amplification were digested with *Aar*I and ligated to the *N*-terminal fusion vector, pBWA(V)HS-ccdb-GLosgfp, for expression driven by the GFP 35S promoter. The GFP overexpression vectors PBWA(V)HS1-3-GLosgfp, PBWA(V)HS2-2-GLosgfp, PBWA(V)HS6-3-GLosgfp were subsequently constructed. The *35S:GFP* vector was selected as the negative control. The constructed vectors were transformed into mesophyll cell protoplasts of *A*. *thaliana* [71]. The GFP signals of the target genes were observed using a laser scanning microscope (Leica TCS SP8, Wetzlar, Germany) with an excitation wavelength of 488 nm. The excitation wavelengths for mKATE and chlorophyll autofluorescence were 561 and 640 nm, respectively. The emission wavelengths for mKATE, GFP, and chlorophyll autofluorescence were 580, 510, and 675 nm, respectively.

### Genetic transformation of *A. thaliana*

Three overexpression vector plasmids, namely PBWA(V)HS1-3-GLosgfp, PBWA(V)HS2-2-GLosgfp, and PBWA(V)HS6-3-GLosgfp, were separately introduced into the GV3101 strain of *Agrobacterium tumefaciens* (syn. *Rhizobium radiobacter*) using the freeze–thaw method. *Arabidopsis thaliana* was transformed using the floral infiltration method [72]. Transformation-positive plants were screened using a medium containing hygromycin. The transgenic homozygous plants of the T3 generation were obtained by multigeneration selfing. The phenotypes of the plants in the T3 generation were observed 14, 28, 35, and 42 days after sowing. The length of the petioles and leaf area were measured using ImageJ software [73].

## Supplementary Information

**Additional file 1: Figure S1.** Comparative analysis of the structural characteristics and expression levels of DElncRNAs and DEmRNAs. (a) Distribution statistics of the lengths of DElncRNA and DEmRNA transcripts. (b) Statistics of the exon numbers of DElncRNA and DEmRNA transcripts. (c, d) Distribution statistics of the ORF lengths of DEmRNA and DElncRNA transcripts. (e) Comparison of the expression levels of DElncRNAs and DEmRNAs.

**Additional file 2: Figure S2.** Co-expression network of DEmRNAs and DElncRNAs.

**Additional file 3: Figure S3.** qRT-PCR analysis of DEmRNAs, DEcircRNAs, DElncRNAs, and DEmiRNAs in the leaves and petioles of Chinese cabbage.

**Additional file 4: Table S1.** Summary of RNA-seq data.

**Additional file 5: Table S2.** Summary of DEmRNAs, DElncRNAs, DEcircRNAs, and DEmiRNAs identified in this study.

**Additional file 6: Table S3.** Annotation and relative expression levels of DEmRNAs involved in leaf and petiole development in Chinese cabbage.

**Additional file 7: Table S4.** List of GO terms for the DEmRNAs, DElncRNAs, and DEmiRNAs.

**Additional file 8: Table S5.** KEGG pathway assignments for the DEmRNAs, DElncRNAs, and DEmiRNAs.

**Additional file 9: Table S6.** Summary of valid data obtained from the small RNA libraries prepared from the RNA-seq data.

**Additional file 10: Table S7.** Summary of ceRNA relationships.

**Additional file 11: Table S8.** Primers used for qRT-PCR.

**Additional file 12: Table S9.** Primers used for cDNA amplification of target genes.

## Data Availability

The datasets supporting the conclusions of this article are included within the article and its additional files. The transcriptome sequencing data were deposited in the National Center for Biotechnology Information (NCBI) SRA database (https://www.ncbi.nlm.nih.gov/sra) under accession number PRJNA906453 and PRJNA899652. Genomic sequences and gene annotation information of *Brassica rapa* were downloaded from http://brassicadb.cn/#/.

## Abbreviations

miRNA: MicroRNA
ncRNA: Non-coding RNA
lncRNA: Long ncRNA
ceRNA: Competitive endogenous RNA
circRNA: Circular RNA
GO: Gene Ontology
KEGG: Kyoto Encyclopedia of Genes and Genomes
qRT-PCR: Quantitative real-time polymerase chain reaction
DEmRNAs: Differentially expressed mRNAs
DElncRNAs: Differentially expressed lncRNAs
DEcircRNAs: Differentially expressed circRNAs
DEmiRNAs: Differentially expressed miRNAs
XTH: Xyloglucan endotransglucosylase/hydrolase
CESA: Cellulose synthase
BZR1: BRASSINAZOLE-RESISTANT1
BES1: BRI1-EMS-suppressor1
GA20OX: Gibberellin 20 oxidase
ORF: Open reading frame
GA: Gibberellin
BR: Brassinosteroid
cMTs: Cortical microtubules
HD-ZIP: Homeodomain leucine zipper
LOB: Lateral organ boundary
