# Supplementary figures and images for: Whole transcriptome analysis and construction of a ceRNA regulatory network related to leaf and petiole development in Chinese cabbage (Brassica campestris L. ssp. pekinensis)
Source: BMC Genomics. 2023 Mar 24;24:144. doi: 10.1186/s12864-023-09239-y (PMC10039531; doi:10.1186/s12864-023-09239-y)

**a**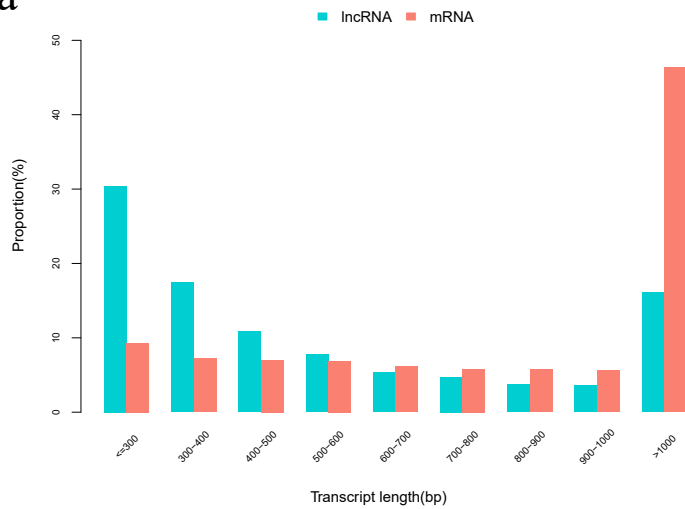**b**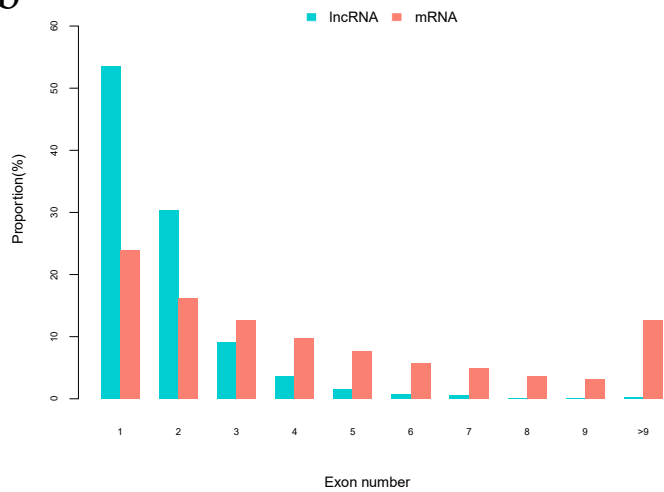**c**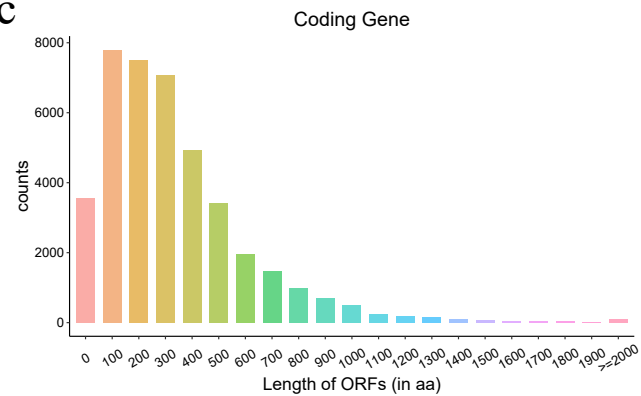**d**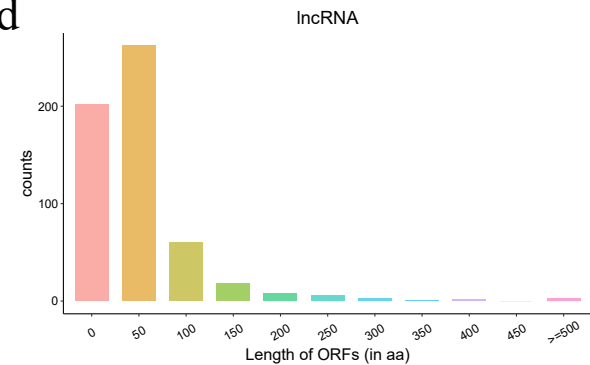**e**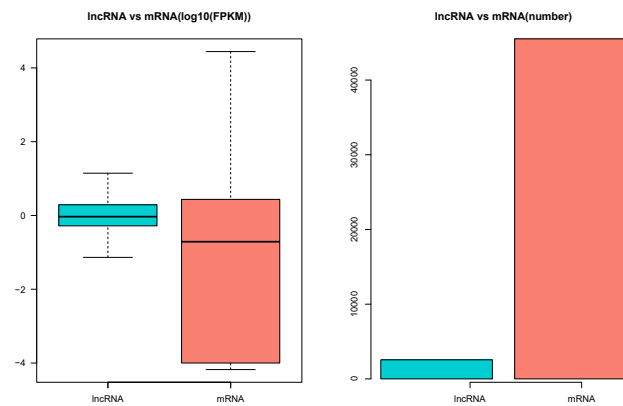

Supplement: Supplementary file 1 — Additional file 1: Figure S1. Comparative analysis of the structural characteristics and expression levels of DElncRNAs and DEmRNAs. (a) Distribution statistics of the lengths of DElncRNA and DEmRNA transcripts. (b) Statistics of the exon numbers of DElncRNA and DEmRNA transcripts. (c, d) Distribution statistics of the ORF lengths of DEmRNA and DElncRNA transcripts. (e) Comparison of the expression levels of DElncRNAs and DEmRNAs. [file 12864_2023_9239_MOESM1_ESM.pdf]

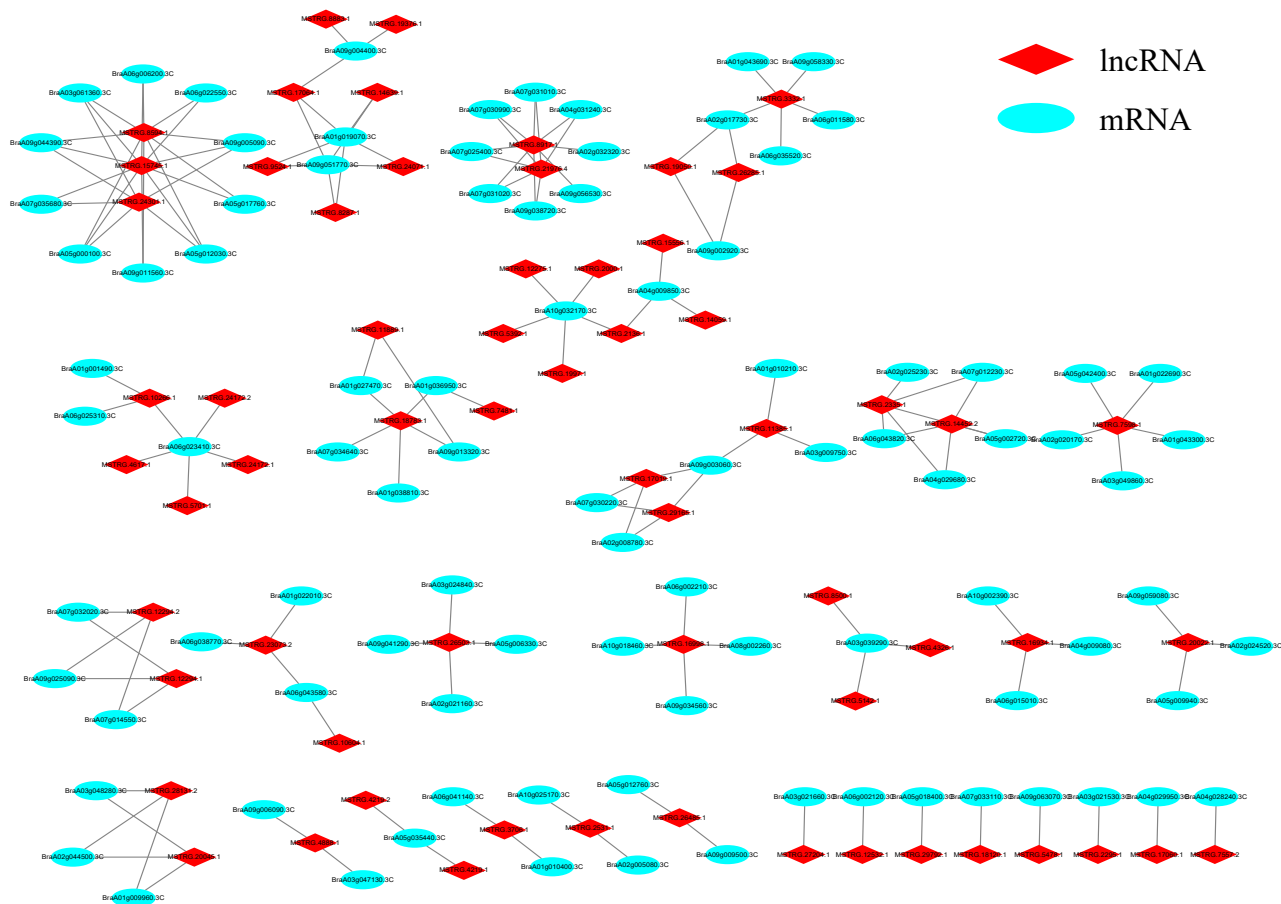

Supplement: Supplementary file 2 — Additional file 2: Figure S2. Co-expression network of DEmRNAs and DElncRNAs. [file 12864_2023_9239_MOESM2_ESM.pdf]

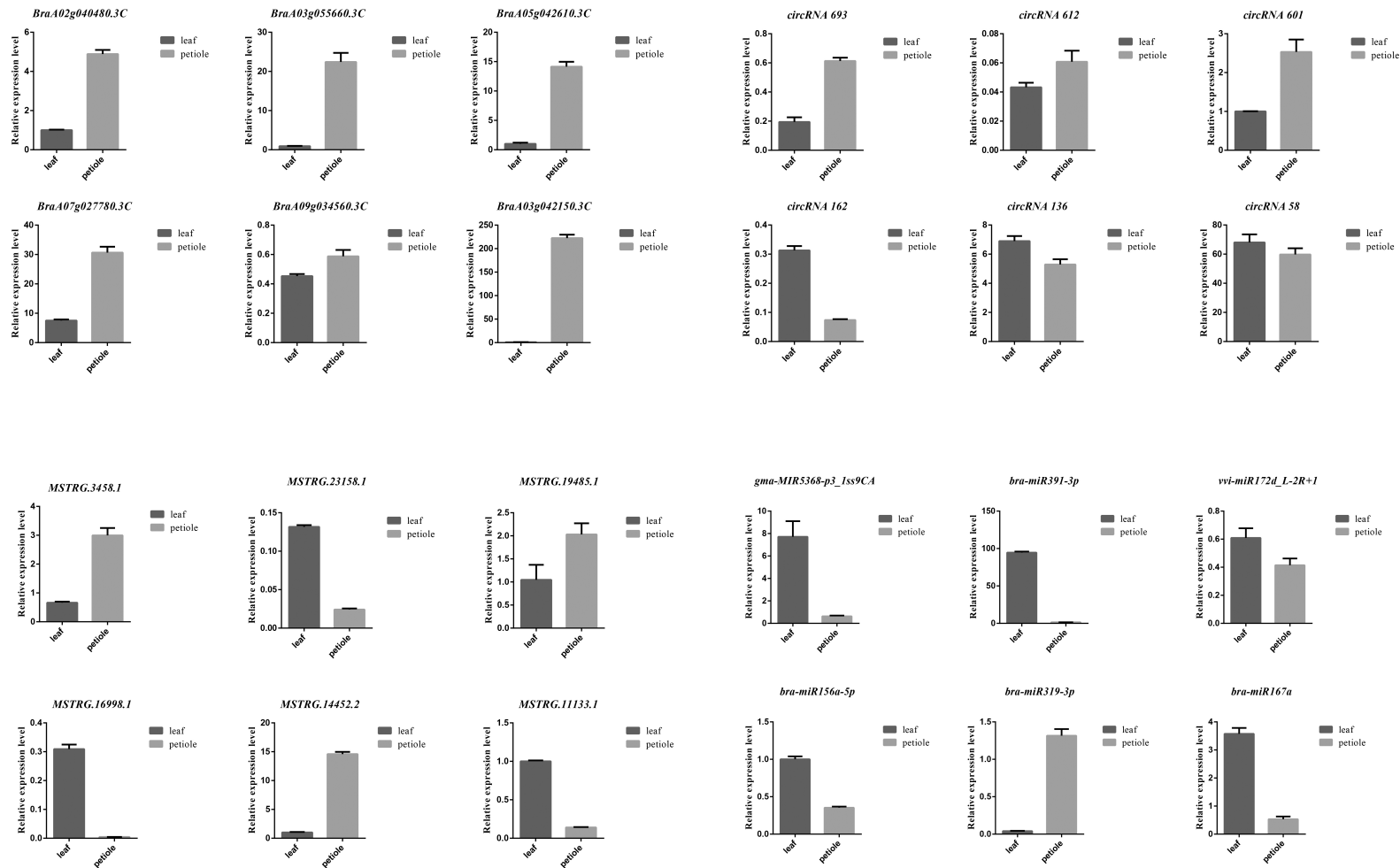

Supplement: Supplementary file 3 — Additional file 3: Figure S3. qRT-PCR analysis of DEmRNAs, DEcircRNAs, DElncRNAs, and DEmiRNAs in the leaves and petioles of Chinese cabbage. [file 12864_2023_9239_MOESM3_ESM.pdf]
