# Supplementary material for: Whole transcriptome analysis and construction of a ceRNA regulatory network related to leaf and petiole development in Chinese cabbage (Brassica campestris L. ssp. pekinensis)
Source: BMC Genomics. 2023 Mar 24;24:144. doi: 10.1186/s12864-023-09239-y (PMC10039531; doi:10.1186/s12864-023-09239-y)
Supplement: Supplementary file 6 — Additional file 6: Table S3. Annotation and relative expression levels of DEmRNAs involved in leaf and petiole development in Chinese cabbage. [file 12864_2023_9239_MOESM6_ESM.docx]

**Table S3** Annotation and relative expression levels of DEmRNAs involved in leaf and petiole development in Chinese cabbage

| Gene ID | FPKM ‘pet’ | FPKM ‘leaf’ | log_2_(Fold change) | Regulation | Annotation term |
| --- | --- | --- | --- | --- | --- |
| *BraA03g055670.3C* | 58.76 | 0.58 | 6.67 | Upregulated | Xyloglucan endotransglucosylase/hydrolase protein 17 |
| *BraA05g008490.3C* | 69.59 | 2.96 | 4.55 | Upregulated | Xyloglucan endotransglucosylase/hydrolase protein 32 |
| *BraA03g042150.3C* | 2450.66 | 90.94 | 4.75 | Upregulated | Xyloglucan endotransglucosylase/hydrolase protein |
| *BraA06g008170.3C* | 57.05 | 1.47 | 5.28 | Upregulated | Xyloglucan endotransglucosylase/hydrolase protein 8 |
| *BraA04g026160.3C* | 72.62 | 2.74 | 4.73 | Upregulated | Xyloglucan endotransglucosylase/hydrolase protein 32 |
| *BraA03g019170.3C* | 94.28 | 6.72 | 3.81 | Upregulated | Xyloglucan endotransglucosylase/hydrolase protein 32 |
| *BraA01g007430.3C* | 160.33 | 8.08 | 4.31 | Upregulated | Xyloglucan endotransglucosylase/hydrolase protein 18 |
| *BraA08g009760.3C* | 36.12 | 122.24 | -1.76 | Downregulated | Xyloglucan endotransglucosylase/hydrolase protein 15 |
| *BraA08g018130.3C* | 12.74 | 0.53 | 4.58 | Upregulated | Xyloglucan endotransglucosylase/hydrolase protein 18 |
| *BraA02g035180.3C* | 8.29 | 21.51 | -1.38 | Downregulated | Xyloglucan endotransglucosylase/hydrolase protein 27 |
| *BraA03g055660.3C* | 348.15 | 55.95 | 2.64 | Upregulated | Xyloglucan endotransglucosylase/hydrolase protein 18 |
| *BraA03g055650.3C* | 101.39 | 24.76 | 2.03 | Upregulated | Xyloglucan endotransglucosylase/hydrolase protein 24 |
| *BraA07g009370.3C* | 12.04 | 4.42 | 1.44 | Upregulated | Xyloglucan endotransglucosylase/hydrolase protein 16 |
| *BraA07g008060.3C* | 5.36 | 2.14 | 1.32 | Upregulated | Xyloglucan endotransglucosylase/hydrolase protein 3 |
| *BraA08g032010.3C* | 3.38 | 0.96 | 1.82 | Upregulated | Xyloglucan endotransglucosylase/hydrolase protein 33 |
| *BraA08g008900.3C* | 5.15 | 2.11 | 1.29 | Upregulated | Xyloglucan endotransglucosylase/hydrolase protein 30 |
| *BraA05g007910.3C* | 73.40 | 2.08 | 5.14 | Upregulated | Expansin-A3 precursor |
| *BraA06g036610.3C* | 13.43 | 0.00 | 17.03 | Upregulated | Expansin-A5 precursor |
| *BraA06g044870.3C* | 171.48 | 11.13 | 3.95 | Upregulated | Expansin-like A2 |
| *BraA09g003690.3C* | 60.18 | 1.94 | 4.96 | Upregulated | Expansin-A5 |
| *BraA06g039430.3C* | 28.71 | 2.56 | 3.49 | Upregulated | Expansin-A15-like precursor |
| *BraA07g000110.3C* | 17.53 | 0.51 | 5.11 | Upregulated | Expansin-B1 precursor |
| *BraA07g015910.3C* | 11.53 | 0.49 | 4.55 | Upregulated | Expansin-A11 |
| *BraA02g035910.3C* | 21.21 | 1.33 | 4.00 | Upregulated | Expansin-A15 |
| *BraA03g021530.3C* | 6.11 | 15.95 | -1.38 | Downregulated | Expansin precursor |
| *BraA04g027880.3C* | 7.88 | 0.33 | 4.56 | Upregulated | Expansin-A4-like |
| *BraA03g047260.3C* | 8.26 | 18.29 | -1.15 | Downregulated | Expansin-like B1 |
| *BraA03g020980.3C* | 35.47 | 2.65 | 3.74 | Upregulated | Expansin-A4 precursor |
| *BraA09g046320.3C* | 0.00 | 1.36 | -13.73 | Downregulated | Expansin-A16 precursor |
| *BraA07g019250.3C* | 36.64 | 9.44 | 1.96 | Upregulated | Expansin-A6 |
| *BraA04g004550.3C* | 0.00 | 1.28 | -13.64 | Downregulated | Expansin-A16-like |
| *BraA02g019240.3C* | 63.02 | 15.43 | 2.03 | Upregulated | Expansin-A1 |
| *BraA05g041750.3C* | 12.48 | 2.15 | 2.54 | Upregulated | Expansin-A13 |
| *BraA01g000070.3C* | 17.79 | 3.23 | 2.46 | Upregulated | Expansin-like A2 |
| *BraA01g043750.3C* | 7.28 | 0.93 | 2.97 | Upregulated | Expansin-A13-like |
| *BraA04g020750.3C* | 28.12 | 5.29 | 2.41 | Upregulated | Expansin-A6 precursor |
| *BraA05g006390.3C* | 16.33 | 2.88 | 2.51 | Upregulated | Expansin-A4-like |
| *BraA10g033350.3C* | 8.64 | 1.10 | 2.97 | Upregulated | Expansin-A9 |
| *BraA07g034440.3C* | 11.47 | 2.71 | 2.08 | Upregulated | Expansin-A1 precursor |
| *BraA01g000050.3C* | 7.31 | 1.56 | 2.23 | Upregulated | Expansin-A20 precursor |
| *BraA01g019010.3C* | 4.98 | 1.01 | 2.31 | Upregulated | Expansin-B3 |
| *BraA03g054290.3C* | 2.85 | 0.58 | 2.30 | Upregulated | Expansin-B3-like precursor |
| *BraA03g000600.3C* | 4.12 | 1.23 | 1.74 | Upregulated | Expansin-A9-like |
| *BraA07g034590.3C* | 18.27 | 1.61 | 3.50 | Upregulated | Transcription factor TCP15-like |
| *BraA02g019350.3C* | 11.42 | 4.67 | 1.29 | Upregulated | Transcription factor TCP15-like |
| *BraA06g043630.3C* | 22.80 | 0.01 | 11.04 | Upregulated | Transcription factor bHLH92 |
| *BraA04g016700.3C* | 17.10 | 0.08 | 7.67 | Upregulated | Transcription factor bHLH19-like |
| *BraA09g001050.3C* | 0.00 | 8.33 | -16.35 | Downregulated | Transcription factor bHLH57 |
| *BraA04g029740.3C* | 1.39 | 13.31 | -3.26 | Downregulated | Transcription factor bHLH130 |
| *BraA05g001310.3C* | 61.46 | 4.17 | 3.88 | Upregulated | Transcription factor ABA-INDUCIBLE bHLH-TYPE |
| *BraA04g030510.3C* | 0.47 | 8.90 | -4.25 | Downregulated | Transcription factor bHLH129-like |
| *BraA09g056320.3C* | 1.01 | 7.05 | -2.81 | Downregulated | Transcription factor bHLH133 |
| *BraA09g018940.3C* | 4.98 | 0.00 | 15.60 | Upregulated | Transcription factor bHLH96-like |
| *BraA09g019940.3C* | 4.48 | 0.00 | 15.45 | Upregulated | Transcription factor bHLH92-like |
| *BraA03g046880.3C* | 41.74 | 7.37 | 2.50 | Upregulated | Transcription factor bHLH3-like |
| *BraA04g032100.3C* | 27.26 | 4.81 | 2.50 | Upregulated | Transcription factor ABA-INDUCIBLE bHLH-TYPE |
| *BraA09g040530.3C* | 4.46 | 0.01 | 9.18 | Upregulated | Transcription factor bHLH94-like |
| *BraA08g034220.3C* | 17.37 | 1.69 | 3.36 | Upregulated | Transcription factor bHLH113-like |
| *BraA05g002760.3C* | 2.66 | 10.34 | -1.96 | Downregulated | Transcription factor bHLH130-like |
| *BraA06g041750.3C* | 0.59 | 4.32 | -2.87 | Downregulated | Transcription factor bHLH71-like |
| *BraA05g002770.3C* | 18.36 | 3.70 | 2.31 | Upregulated | Transcription factor bHLH48 |
| *BraA07g036580.3C* | 2.58 | 6.08 | -1.24 | Downregulated | Transcription factor bHLH96-like |
| *BraA10g000650.3C* | 27.42 | 6.72 | 2.03 | Upregulated | Transcription factor bHLH13 |
| *BraA05g012920.3C* | 5.37 | 0.33 | 4.01 | Upregulated | Transcription factor bHLH113 |
| *BraA06g035500.3C* | 4.40 | 0.68 | 2.69 | Upregulated | Transcription factor bHLH78 |
| *BraA07g014650.3C* | 1.06 | 0.00 | 13.37 | Upregulated | Transcription factor bHLH94-like |
| *BraA03g046740.3C* | 12.88 | 1.47 | 3.13 | Upregulated | Transcription factor bHLH147-like |
| *BraA05g025700.3C* | 0.60 | 2.72 | -2.19 | Downregulated | Transcription factor bHLH87 |
| *BraA02g045520.3C* | 0.03 | 2.99 | -6.83 | Downregulated | LOB domain-containing protein 37-like |
| *BraA07g017050.3C* | 1.73 | 0.10 | 4.15 | Upregulated | LOB domain-containing protein 36 |
| *BraA02g037830.3C* | 5.60 | 0.32 | 4.12 | Upregulated | LOB domain-containing protein 25-like |
| *BraA03g044950.3C* | 11.22 | 2.92 | 1.94 | Upregulated | LOB domain-containing protein 38 |
| *BraA01g001340.3C* | 11.30 | 3.69 | 1.62 | Upregulated | LOB domain-containing protein 39 |
| *BraA05g014110.3C* | 5.23 | 0.13 | 5.34 | Upregulated | LOB domain-containing protein 12 |
| *BraA02g016770.3C* | 3.74 | 0.64 | 2.54 | Upregulated | LOB domain-containing protein 6 |
| *BraA03g060820.3C* | 2.90 | 0.51 | 2.50 | Upregulated | LOB domain-containing protein 39-like |
| *BraA07g018870.3C* | 4.25 | 0.30 | 3.80 | Upregulated | LOB domain-containing protein 11-like |
| *BraA07g010160.3C* | 2.71 | 0.67 | 2.01 | Upregulated | LOB domain-containing protein 4 |
| *BraA09g041230.3C* | 4.93 | 1.91 | 1.37 | Upregulated | LOB domain-containing protein 38-like |
| *BraA01g002030.3C* | 8.97 | 58.94 | -2.72 | Downregulated | BES1/BZR1 homolog protein 2 |
| *BraA07g041760.3C* | 5.71 | 0.06 | 6.60 | Upregulated | BES1/BZR1 homolog protein 4 |
| *BraA02g024860.3C* | 19.57 | 6.91 | 1.50 | Upregulated | BES1/BZR1 homolog protein 4 |
| *BraA10g030020.3C* | 0.19 | 6.18 | -5.04 | Downregulated | Gibberellin 20 oxidase 3 |
| *BraA10g010400.3C* | 1.59 | 4.86 | -1.61 | Downregulated | Gibberellin 20 oxidase 2 |
| *BraA09g023210.3C* | 124.46 | 59.93 | 1.05 | Upregulated | RGA-like protein 2 |
| *BraA02g017030.3C* | 28.21 | 9.31 | 1.60 | Upregulated | DELLA protein RGL1 |
| *BraA10g022510.3C* | 7.36 | 2.88 | 1.35 | Upregulated | DELLA protein RGL3 |
| *BraA01g014690.3C* | 0.00 | 4.24 | -15.37 | Downregulated | Cellulose synthase-like protein G2 |
| *BraA01g000390.3C* | 18.91 | 0.37 | 5.67 | Upregulated | Cellulose synthase A catalytic subunit 2 |
| *BraA10g001400.3C* | 10.55 | 0.99 | 3.42 | Upregulated | Cellulose synthase-like protein D5 |
| *BraA01g043890.3C* | 18.64 | 0.76 | 4.62 | Upregulated | Cellulose synthase-like protein D3 |
| *BraA03g002020.3C* | 175.07 | 25.53 | 2.78 | Upregulated | Cellulose synthase A catalytic subunit 3 |
| *BraA05g016030.3C* | 0.94 | 3.03 | -1.69 | Downregulated | Cellulose synthase-like protein E1 |
| *BraA05g041600.3C* | 25.12 | 4.97 | 2.34 | Upregulated | Cellulose synthase-like protein D3 |
| *BraA06g028190.3C* | 174.83 | 37.44 | 2.22 | Upregulated | Cellulose synthase A catalytic subunit 6 |
| *BraA04g023370.3C* | 0.10 | 1.40 | -3.87 | Downregulated | Cellulose synthase-like protein B3 |
| *BraA04g023360.3C* | 0.00 | 0.32 | -11.67 | Downregulated | Cellulose synthase-like protein B3 |
| *BraA02g001600.3C* | 229.26 | 53.32 | 2.10 | Upregulated | Cellulose synthase A catalytic subunit 3 |
| *BraA03g048470.3C* | 7.57 | 1.39 | 2.44 | Upregulated | Cellulose synthase A catalytic subunit 8 |
| *BraA03g004030.3C* | 111.77 | 27.72 | 2.01 | Upregulated | Cellulose synthase A catalytic subunit 5 |
| *BraA03g008010.3C* | 4.97 | 1.14 | 2.12 | Upregulated | Cellulose synthase A catalytic subunit 7 |
| *BraA01g005650.3C* | 172.85 | 45.40 | 1.93 | Upregulated | Cellulose synthase A catalytic subunit 1 |
| *BraA03g051590.3C* | 0.90 | 2.14 | -1.26 | Downregulated | Cellulose synthase-like protein G3 |
| *BraA06g043400.3C* | 7.12 | 1.53 | 2.22 | Upregulated | Cellulose synthase A catalytic subunit 4 |
| *BraA10g022900.3C* | 147.46 | 48.66 | 1.60 | Upregulated | Cellulose synthase-like protein D2 |
| *BraA02g007030.3C* | 0.41 | 0.04 | 3.51 | Upregulated | Cellulose synthase A catalytic subunit 7 |
| *BraA02g006730.3C* | 2.40 | 0.80 | 1.58 | Upregulated | Cellulose synthase-like protein D2 |
| *BraA03g018520.3C* | 23.13 | 8.21 | 1.49 | Upregulated | Protein MOR1-like |
